# Preparation of Silica Nanoparticles Loaded with Nootropics and Their *In Vivo* Permeation through Blood-Brain Barrier

**DOI:** 10.1155/2015/812673

**Published:** 2015-05-17

**Authors:** Josef Jampilek, Kamil Zaruba, Michal Oravec, Martin Kunes, Petr Babula, Pavel Ulbrich, Ingrid Brezaniova, Radka Opatrilova, Jan Triska, Pavel Suchy

**Affiliations:** ^1^Faculty of Pharmacy, University of Veterinary and Pharmaceutical Sciences Brno, Palackeho 1/3, 612 42 Brno, Czech Republic; ^2^Faculty of Chemical Engineering, University of Chemistry and Technology Prague, Technicka 5, 166 28 Prague 6, Czech Republic; ^3^Global Change Research Centre AS CR, Belidla 986/4a, 603 00 Brno, Czech Republic; ^4^Faculty of Food and Biochemical Technology, University of Chemistry and Technology Prague, Technicka 5, 166 28 Prague 6, Czech Republic

## Abstract

The blood-brain barrier prevents the passage of many drugs that target the central nervous system. This paper presents the preparation and characterization of silica-based nanocarriers loaded with piracetam, pentoxifylline, and pyridoxine (drugs from the class of nootropics), which are designed to enhance the permeation of the drugs from the circulatory system through the blood-brain barrier. Their permeation was compared with non-nanoparticle drug substances (bulk materials) by means of an *in vivo* model of rat brain perfusion. The size and morphology of the nanoparticles were characterized by transmission electron microscopy. The content of the drug substances in silica-based nanocarriers was analysed by elemental analysis and UV spectrometry. Microscopic analysis of visualized silica nanocarriers in the perfused brain tissue was performed. The concentration of the drug substances in the tissue was determined by means of UHPLC-DAD/HRMS LTQ Orbitrap XL. It was found that the drug substances in silica-based nanocarriers permeated through the blood brain barrier to the brain tissue, whereas bulk materials were not detected in the brain.

## 1. Introduction

Nootropics are a wide and structurally heterogeneous class of drugs (also supplements, nutraceuticals, and functional foods) that improve one or more aspects of mental function, such as working memory, motivation, and attention. They can be also referred to as smart drugs, memory enhancers, neuroenhancers, cognitive enhancers, and intelligence enhancers. Their therapeutic effect is based on positive affection of metabolic pathways in brain tissue (improved utilization of nutrients and mediators) and their impact manifests after some time of administration. They are used especially at insult of brain by a trauma, ischemia, intoxication, and hypoxia as well as at neurodegenerative disorders such as Alzheimer's disease, Parkinson's disease, Huntington's disease, and attention deficit hyperactivity disorder (ADHD). A number of nootropics are synthetic analogues of physiological compounds (such as acetylcholine, pyridoxine, GABA, or coenzyme Q_10_); others are natural compounds (e.g., vinpocetine); and the rest are other cerebral-active compounds (e.g., nimodipine, pentoxifylline, etc.) [1, 2].

The site of action of all these drugs is brain; that is, they must overcome all barriers to achieve the brain tissue, and the blood-brain barrier (BBB) is the last, critical, and serious obstacle for the permeation of drugs that require CNS action. The BBB represents a structure with complex cellular organisation that separates the brain parenchyma from the systemic circulation. It consists of brain capillaries that support endothelial cells and are surrounded by astrocytic end-foot processes. The BBB also acts as a metabolic barrier due to the presence of numerous enzymes. These enzymes can either metabolise potentially harmful drugs to CNS-inactive compounds or convert inactive drugs to their active CNS metabolites or degrade them into metabolites or substrates of specific efflux transporters, such as P-glycoprotein/multidrug resistance proteins [3–5].

All the above mentioned properties of the barrier result in strong selection of permeating drugs depending on their physicochemical properties, such as molecular weight, molecular volume, lipophilicity, ionisation state, and/or their affinity to specific transporters (uptake/efflux transporters) [3, 5]. The cellular organisation of the BBB and the presence of transmembrane proteins enable a selective regulation of the passage of molecules from the blood to the brain. Molecules present in the blood stream can reach the CNS by two different pathways, the paracellular pathway (through tight junctions) and the transcellular pathway (through endothelial cells). Molecules that reach the CNS via the transcellular pathway can diffuse passively, be actively transported by specific transporters, or undergo endocytosis [5, 6]. To circumvent the BBB and allow an active CNS compound to reach its target, many strategies exist. They can be sorted with respect to the BBB as either invasive (direct injection into the cerebrospinal fluid or therapeutic opening of the BBB) or noninvasive such as use of alternative routes of administration (e.g., nose-to-brain route and olfactory and trigeminal pathways to brain), inhibition of efflux transporters, chemical modification of drugs (prodrugs and bioprecursors), and encapsulation of drugs into nanocarriers (e.g., liposomes, polymeric nanoparticles, and solid lipid nanoparticles) [5, 7]. Nanoparticles as drug carriers have also been extensively studied recently. Their uptake into the brain is hypothesised to occur via adsorptive transcytosis and receptor-mediated endocytosis [8, 9]. Particle size, surface affinity, and stability in circulation are important factors influencing the brain distribution of colloidal particles [10, 11].

Silica-based nanoparticles are widely used in nanotechnology in the biomedical sector, because they are easy to prepare and inexpensive to produce. Their specific surface characteristics, porosity, and capacity for functionalization make them good tools for biomolecule detection and separation, providing solid media for drug delivery systems and for contrast agent protectors. In addition, they are used as safe and biocompatible pharmaceutical additives [12–17]. Incorporation of a drug into nanocarriers may change the drug bioavailability, physicochemistry, and pharmacokinetics, which can be advantageous in many applications [18, 19].

**Figure 1:**
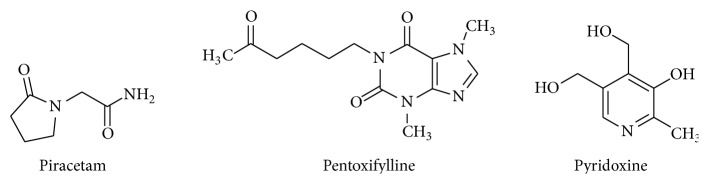
Structures of investigated drugs.

As mentioned above, silica-based nanoparticles are well-known to be biocompatible easy-to-prepare nontoxic carriers that are able to transport loaded drugs in living organisms [12–17]. What is not so well-known (only a few papers have been published so far), they are also able to penetrate through BBB which is used for transport of silica nanoparticles [20] and silica-coated nanoparticles [21]. The aim of this study was the preparation of these silica-based nanocarriers loaded with piracetam, pentoxifylline, and pyridoxine (see Figure 1) and investigation of their permeation through the BBB in comparison with bulk drug substances to enhance absorption and concentration of these cerebral-active drugs in brain.

## 2. Experimental and Methods

### 2.1. General

All reagents were purchased from Sigma-Aldrich, Life Technologies, or Fisher Chemical and were of analytical grade. Acetonitrile hypergrade for LC-MS LiChrosolvR was supplied by Merck KGaA (Darmstadt, Germany). Methanol hypergrade for LC-MS LiChrosolvR was supplied by Merck KGaA (Darmstadt, Germany). Acetic acid was obtained from Sigma-Aldrich Chemie GmbH (Steinheim, Germany). The Purelab Classic (ELGA LabWater, High Wycombe, Bucks, UK) was used to generate high purity water for preparation of aqueous mobile phase.

### 2.2. Preparation of Nanoparticles

The silica nanoparticles were made via a modified Stober method [22–25]. The typical reaction solution consisted of methanol (99.9%, 94 mL), ammonium hydroxide (25% wt of ammonia, 30 mL), and drug solution (20 mg/mL for piracetam, 5–20 mg/mL for others, 2 mL). On mixing the solution by vigorous magnetic stirring, tetraethylorthosilicate (TEOS) (99.9%, 0.8 mL) was added dropwise to initiate the hydrolysis reaction. The resulting solution was stirred at room temperature for 2 h. The particle suspension was repeatedly (4 times) collected by centrifugation (10 min, 9,000 G) and washed with methanol to ensure the removal of all unreacted reactants. Finally, the nanoparticles were dried to yield a final product of drug-loaded silica nanocarriers.

### 2.3. Elemental Analysis and UV Spectrometry

The content of API (1.5 mg/50 mg nanoparticles) in silica nanoparticles was determined by elemental analysis and UV absorption. Elemental analysis was performed using a Vario EL III Universal CHNOS Elemental Analyzer (Elementar Analysensysteme, Germany) and loading was calculated from the corresponding increase of nitrogen content when drug-loaded nanoparticles were analysed in comparison with nonloaded nanoparticles. Calculated loadings for pentoxifylline and pyridoxine were in accordance with those estimated by UV absorption by determination of unbound drug removed by nanoparticle washing. The corresponding verification of piracetam loading estimated by elemental analysis was not possible due to piracetam absorption below 210 nm. Absorption measurements were performed using a Cintra 404 spectrometer (GBC Scientific Equipment, USA).

### 2.4. Transmission Electron Microscopy

**Figure 2:**
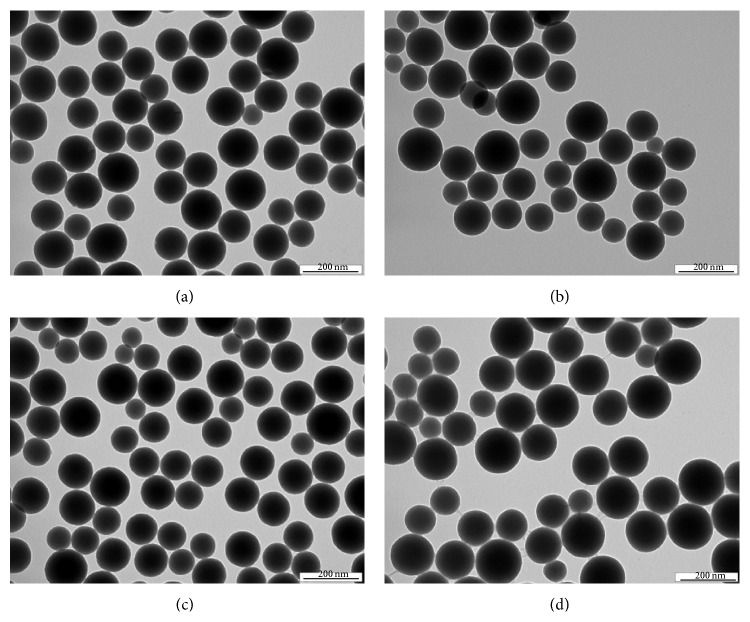
TEM microphotographs of pure silica nanoparticle (a), silica-based nanocarriers loaded with piracetam (b), pentoxifylline (c), and pyridoxine (d).

**Figure 3:**
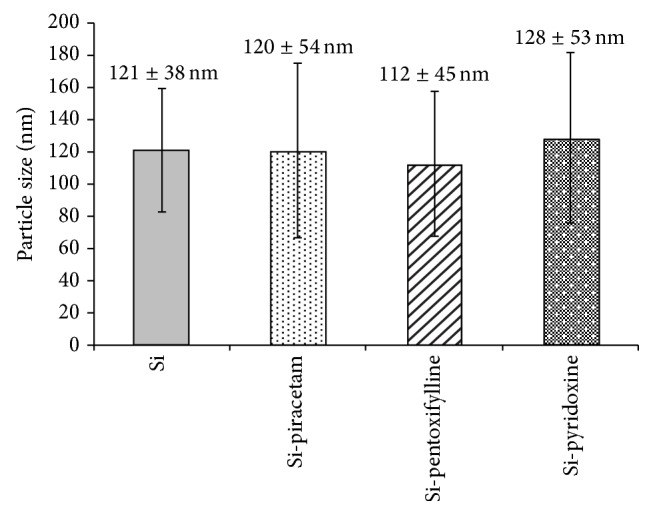
Particle size [nm] of individual nanoparticles: pure silica nanoparticles, Si-piracetam, Si-pentoxifylline, and Si-pyridoxine. Particle size is expressed as mean diameter ± 2∗SD (*n* > 50 particles).

The particle size and the morphology of the samples were examined by transmission electron microscopy (TEM). Samples for TEM were prepared by putting a drop of the colloidal dispersion in methanol (10 *μ*L) on a copper grid covered with thin amorphous carbon film. Samples were dried before inserting them in the specimen holder of a transmission electron microscope JEOL JEM-1010 and observed at 80 kV. Pictures were taken by a digital camera SIS Megaview III (Soft Imaging Systems) and analysed by AnalySIS 2.0 software. The average particle size was calculated from at least 50 particles. The results are illustrated in Figures 2 and 3.

### 2.5. Rat Brain Perfusion

Male Wistar rats (310–380 g) purchased from the breeding facility Anlab (Prague, Czech Republic) were used in the study. Animals were maintained under standard conditions of temperature and lighting and given food and water* ad libitum*. For surgical preparation, rats were anesthetized intramuscularly with ketamine and xylazine. The in situ rat brain perfusion technique was used with modifications according to the previously described methods [26–28]. External jugular veins were prepared and cannulated for freely blood flowing out of the veins. At the same time, carotid arteries (on the left and right sides) were prepared and cannulated using intravascular catheter filled with heparinized saline (60 U/mL) for perfusion. Ligation was accomplished caudally to the catheter implantation site. The catheter in the carotid artery was connected to a syringe filled with buffered Krebs-Henseleit saline solution containing NaCl (7.48 g/L), NaHCO_3_ (2.02 g/L), KCl (0.31 g/L), NaH_2_PO_4_·2H_2_O (0.37 g/L), CaCl_2_ (0.166 g/L), MgCl_2_·6H_2_O (0.18 g/L), and d-glucose 1.803 g/L, as also used in previous perfusion studies [29, 30]. Polyvinylpyrrolidone (35 g/L) was added into the perfusate to maintain physiological oncotic pressure in the perfusion medium. The perfusion fluid was filtered, warmed to 37°C, and gassed with 95% O_2_ and 5% CO_2_. Immediately prior to the perfusion the pH and osmolarity of this solution were 7.35 and 290 mOsm, respectively. The perfusion fluid was infused into the carotid artery with an infusion pump for 240 s at flow rate 10.4 mL/min. This perfusion rate was selected to maintain the carotid artery pressure of 120 mmHg [26]. The rectal temperature of the animal was maintained at 37 ± 0.5°C throughout the surgery by a heat pad connected to a feedback device. At the end of perfusion, rats were decapitated and the whole brain was removed from the skull. Cerebral hemispheres were dissected and stored to next analysis after removal of the arachnoid membrane and meningeal vessels (deeply frozen at −80°C for HPLC analysis and tissues for histological examination were fixed in 10% formaldehyde).

### 2.6. Microscopic Analysis of Brain Tissue: Visualization of Silicates

**Figure 4:**
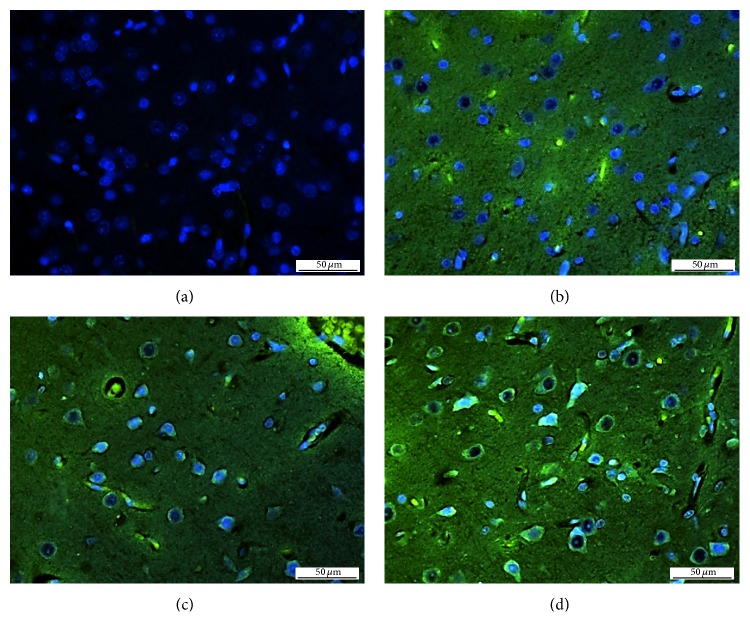
Microscopic photographs of histological preparations of control rat brain tissue (a) and rat brain tissues treated with Si-piracetam (b), Si-pentoxifylline (c), and Si-pyridoxine (d) as stained with PDMPO (green fluorescence) in combination with Hoechst 33258 (nuclei, blue fluorescence).

The deposition of silicates in brain tissue was visualized using *N*-[2-(dimethylamino)ethyl]-2-{4-[2-(pyridin-4-yl)-1,3-oxazol-5-yl]phenoxy}acetamide (PDMPO). The modified procedure described by Shimizu et al. was used [31]. Briefly, sections were deparaffinised and rehydrated (xylene, mixture xylene/ethanol 1 : 1, ethanol, 95% ethanol, 70% ethanol, 50% ethanol, Na-phosphate buffer (0.1 M, pH 7.0)). Then the sections were incubated in 1 *μ*M solution of PDMPO in 0.1 M Na-phosphate buffer (pH 7.0) for 3 hours at 22°C (laboratory temperature). After incubation, the sections were costained with a Hoechst 33258 fluorescent probe (Sigma-Aldrich, USA) to visualize nuclei washed three times with PBS buffer and observed with a microscope using an appropriate excitation filter (Axioscop 40, Zeiss, Germany). Typical photographs are shown in Figure 4.

### 2.7. Analysis of Brain Tissue

Samples of brain tissue after perfusion with bulk materials (non-nanoparticle drug substances) and drug-loaded silica nanocarriers were frozen and homogenized before extraction. Solid-liquid extraction in methanol was used as an efficient method. Extracts were subsequently purified, concentrated, and analysed. The samples of brain tissue (without purification) perfused with piracetam were also analysed by direct injection (overdosing loop) on UHPLC-HRMS. Direct injection was used for the samples where the concentration of the drug was close to or lower than the limit of detection or quantification. Direct injection was not useful for more samples because it can contaminate UHPLC-HRMS.

Isolated drugs from brain tissue and from silica nanoparticles were analysed by a UHPLC-HRMS separation system (Dionex UltiMate 3000 Liquid Chromatography Systems) equipped with diode array detection (DAD) and a hybrid high resolution mass spectrometer LTQ Orbitrap XL (ThermoFisher Scientific, USA/Dionex RSLC, Dionex, USA). A chromatographic column Hypersil Gold (Thermo Scientific, USA), C18 3 *μ*m, 2.1 × 50 mm, was used. The mixture of MeCN-HPLC grade (20.0%) and H_2_O-HPLC grade with 0.1% AcOH (80.0%) was used as a mobile phase. The total flow of the column was 0.3 mL/min, column temperature was 30°C, and the time of analysis was 15 min.

The records were evaluated from the DAD and HRMS-Orbitrap. The wavelengths of 254 nm, 272 nm, 274 nm, 331 nm, and 190–800 nm were monitored. MS and MS^n^ were performed using the HRMS LTQ Orbitrap XL (ThermoFisher Scientific, USA) equipped with a HESI II (heated electrospray ionization) source. Orbitrap was operated in full scan with resolution 60,000. Full scan spectra were acquired over mass range *m*/*z* 50–1000 in the positive mode. Orbitrap was also operated in SIM (select ion monitoring): 143.0810 (as a qualifier ion) and 126.0548 (as a qualifier ion without NH_2_ group) under 1 Δppm for piracetam; 279.1461 under 1 Δppm for pentoxifylline; and 170.0810 (as a qualifier ion) and 152.0700 (as qualifier ion without OH group) under 1 Δppm for pyridoxine. The resolution and sensitivity of Orbitrap were controlled by injection of the standard (piracetam, pentoxifylline, and pyridoxine) after analysing of every 5 samples, and the resolution was also checked by the help of lock masses (phthalates). Blanks were also analysed within the sequence after analysis of each sample. The compounds were checked in the mass library that was created from measurement of standards of piracetam and pentoxifylline and pyridoxine in the MS and MS^n^ modes of Orbitrap.

## 3. Results and Discussion

The content of drug substances in silica-based nanocarriers was analysed by elemental analysis and confirmed by UV absorption analysis of supernatants (except of piracetam). Loading efficiency was tested by the addition of an increasing concentration (5, 10, and 20 mg/mL) of drug solution to the reaction mixture for nanoparticle preparation. For both compounds (pentoxifylline and pyridoxine) the loading efficiency was about 10% for concentration of drug stock solution 10 and 20 mg/mL. When 5 mg/mL drug stock solution was added, the amount of the drug loaded was below the detection limit of the used method. Approximately the same loading efficiency (i.e., 10%) was confirmed for all three drugs by elemental analysis. Only nanoparticles with the highest loading (i.e., those prepared using a drug stock solution of 20 mg/mL) were subjected to the animal study. It was found that 1.5 mg of drug substance is in 50 mg of nanoparticles with batch to batch variance less than 10%. The particle size and the shape of prepared silica nanocarriers with loaded piracetam, pentoxifylline, and pyridoxine (Si-piracetam, Si-pentoxifylline, and Si-pyridoxine) were measured by TEM (see Figure 2). A control sample of pure silica nanoparticles was prepared and characterised. It was found that the average particle size of all prepared nanoparticles was approximately 120 nm (TEM microphotographs of pure silica nanoparticles and nanocarriers with loaded drugs in Figure 2 are shown with 10^5^x magnification). It is evident that the general shape of all particles can be considered as spherical. Based on 95% confidence interval computed from the mean diameter plus or minus* twice* the standard deviations (see Figure 3), it can be stated that no statistical significance was found for the samples.

Microscopic photographs of histological preparations (samples of brain tissue after perfusion with bulk drug solution and Si-drugs) are shown in Figure 4. In comparison with control rat brain tissue (Figure 4(a)), significant changes in fluorescence in silica-based nanocarriers were recorded for piracetam (Figure 4(b)), pentoxifylline (Figure 4(c)), and pyridoxine (Figure 4(d)). In these photographs, both nuclei and cytoplasmic structures have been identified as PDMPO-positive (green fluorescence). No PDMPO fluoresce has been identified in control, untreated samples. In addition, also tissue treated with bulk material alone was investigated. In this case, no changes in fluorescence (not shown) were observed. PDMPO (Lysosensor DND-160 Yellow/Blue) was originally developed to visualize acidic compartments in the cells [32]. On the other hand, it has been established that it has unique properties allowing to evaluate the deposition of silica in cells and tissues. PDMPO-Si complex possesses unique fluorescent properties in the presence of silicic acid, producing bright green fluorescence after UV excitation. PDMPO has successfully been established to visualize silica in diatoms and other organisms. For example, Znachor et al. used PDMPO to study the deposition of silica in* Fragilaria crotonensis *Kitton [33]. This compound was also studied for the distribution and deposition of silicates in horsetail,* Equisetum arvense *L. Shimizu et al. showed on the silica gel that PDMPO is able to label silicates directly [31]. In the light of this fact, it was decided to use it as a probe to visualize deposition of silica-based nanoparticles in tissues.

The samples of brain tissue after perfusion with bulk materials (drugs solutions) and drug-loaded silica nanocarriers were analysed by direct injection (over dosing loop) on UHPLC-HRMS. Also extracted drug substances from the samples of the perfused tissues were analysed. While the bulk drug substances were extracted from perfused brain tissues easily practically by any of the applied methods, the extraction of the nanonized drug substances from the tissues or from silica nanocarriers was problematic. Different extraction techniques for isolation of the drugs from the tissue and the nanocarriers were tested, such as classical liquid extraction (LE), sequent extraction by various solvents (methanol, water, acetonitrile, etc.), solid-liquid extraction, accelerated solvent extraction (ASE), and ultrasound extraction (USE). The individual extraction methods were compared. The used methods showed similar effectivity; nevertheless the solid-liquid extraction in methanol with minimum losses of the studied compounds was selected.

**Figure 5:**
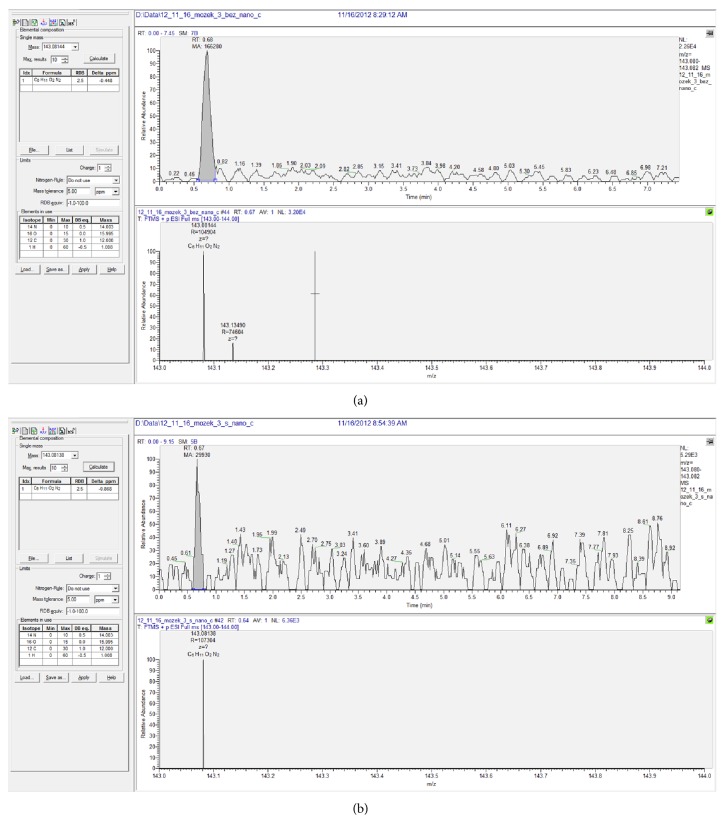
Comparison of concentration of bulk piracetam (a) and Si-piracetam (b) in brain tissue samples: this chromatogram presents very low concentration of drug, close to limit of quantification (S/N close 20/1). Found ratio of piracetam/Si-piracetam is approximately 20 : 1.

For example, the brain tissue samples with piracetam and Si-piracetam were compared. Concentrations of piracetam in brain tissues measured by the UHPLC-HRMS using individual extraction methods were comparable (4–10 ng/mL, 10–40 ng/g brain tissue). For Si-piracetam concentrations measured by individual extraction methods were also comparable (50 pg/mL–1 ng/mL, 100 pg/g brain tissue–2.5 ng/g brain tissues) but with lower extraction efficiency. The extraction efficiency was especially influenced by sorption of the drug substances in silica-based nanocarriers. The determined concentrations in the samples of bulk piracetam were higher than in the brain tissue samples with nanoparticles; see Figure 5. The fact of the strong sorption of piracetam in silica nanocarriers was confirmed using direct injection of the brain tissue with the nanoparticles into the dosing loop and subsequent multiple rinsing by solvent (methanol) and analysis by LC-MS (as mentioned below). Processing of extracts (filtration and centrifugation) caused high losses; nevertheless the highest losses were observed at purification of the extracts (30–40%), because nanocarriers were adsorbed in the filters and on the vial wall. Therefore, silica-based nanoparticles were destroyed by borate buffer (pH = 10.5), but at following LC-MS analysis various borate adducts and dimers were determined; that is, the method does not seem to be suitable.

**Figure 6:**
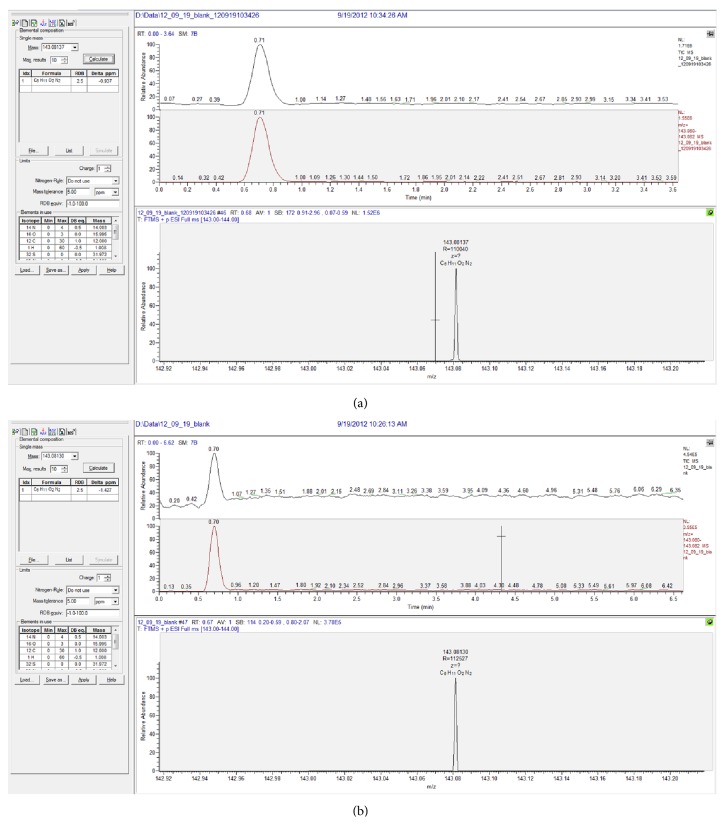
Chromatograms after first (a) and second (b) injections of pure methanol to sample of brain tissue with piracetam-loaded silica nanocarriers (labelled samples as blank-pure solvent injected into sample of brain with nanoparticles.).

As a solution of the above mentioned problems (sorption, dimers, etc.), the samples were analysed by direct injection to a sampling loop with subsequent multiple elution by MeOH. The concentration of Si-piracetam determined by direct injection was by several orders of magnitude higher compared with the concentrations determined in the extracts. By repeated injection of pure methanol the nanocarriers were gradually disintegrated and Si-piracetam was released, as illustrated in chromatograms in Figure 6. Concentrations determined at the first injection of pure solvent was 1500–1000 ng/mL (375–250 ng/mL in 100 mg brain tissue), at the second injection 250–100 ng/mL (65–25 ng/mL in 100 mg brain tissue), which was by 2-3 orders of magnitude higher than those measured during the extraction of brain tissue with nanoparticles.

Based on this semiquantitative method of direct injection, the concentrations of Si-piracetam that permeated through the BBB to the brain can be determined in comparison with drugs in bulk material; see Table 1. The concentration of the permeated bulk piracetam in the brain tissue was 2.8–1.0 ng/mL in 100 mg brain tissue, while that of Si-piracetam was 440–275 ng/mL in 100 mg brain tissue; that is, the application of nanoparticles led to an increase of piracetam approximately 200-fold. Similar strong sorption in silica nanocarriers can be found for pentoxifylline and pyridoxine; nevertheless, as mentioned in Section 2.7, the direct injection of the pentoxifylline samples was not performed due to the contamination of UHPLC-HRMS system. However, based on the results obtained for piracetam, it can be supposed that the concentration of Si-pentoxifylline in the brain tissue would be much higher, approximately 44000–20600 ng/mL in 100 mg of brain tissue. Although the silica nanocarriers loaded with pyridoxine were detected by microscopic analysis of brain tissue, no pyridoxine was found by UHPLC-HRMS; see Table 1.

It can be stated that loading of drugs to silica nanocarriers and extraction of drugs from nanocarriers is governed by general principles of normal-phase adsorption chromatography; it means that the retention of a molecule (the strength of interactions between the molecule and silica surface/silanol groups) is determined by its polar functional groups/double bonds and steric factors. Silica gel has acidic properties, and, therefore, basic compounds interact with the surface of this gel strongly [34]. Thus the observed strong binding of the discussed drug substances in silica nanocarriers may be caused by the presence of free electron pairs in the compounds (basicity). The basicity of pyridoxine expressed as the strongest p*K*_a_(base) was 5.0 ± 0.1 (predicted by ACD/Percepta ver. 2012), while for pentoxifylline the strongest p*K*_a_(base) was 0.5 ± 0.7 (ACD/Percepta ver. 2012) and for piracetam the strongest p*K*_a_(base) was −0.6 ± 0.2 (ACD/Percepta ver. 2012). Based on these data, pyridoxine shows the strongest potential bonding power, which may be a reason why it was not extracted in a detectable amount.

## 4. Conclusions

The silica-based nanocarriers loaded with piracetam, pentoxifylline, and pyridoxine were prepared. The content of the drug substances in silica-based nanocarriers was determined by elemental analysis and spectrophotometry as 1.5 mg of drug in 50 mg of nanoparticles. By transmission electron microscopy it was found that the average particle size of all prepared nanoparticles was approximately 120 nm, and they had spherical shape. The permeation of the prepared nanoparticles was compared with bulk materials by means of the* in vivo* model of rat brain perfusion. Samples of rat brain tissues were analysed by microscope, and it was found that all silica-based nanoparticles permeated to the brain tissue. The concentration of the drug substances in the tissue was determined by LC-HRMS. It was found that all the drugs exhibited very strong sorption in silica nanocarriers. The direct injection of samples of brain tissue (without purification) treated with Si-piracetam to a sampling loop with subsequent multiple elution by MeOH confirmed approximately 200-fold higher concentration of piracetam loaded in silica-based nanocarriers in the brain tissue in comparison with bulk piracetam.

The field of nanomedicine proposes many opportunities of finding novel solutions to improve health care. This study confirmed that silica nanoparticles can permeate through the blood-brain barrier and effectively transport drugs to the brain and so help in the treatment of different difficult to treat cerebral diseases. However, further investigation and, primarily, selection of suitable drug candidates (bulky and nonbasic) for immobilization into silica nanoparticle drug formulations are needed.

## Figures and Tables

**Table 1:** Concentrations of drug-loaded silica nanocarriers permeated through the BBB to brain in comparison with drugs in bulk (n.d. = not detected).

Drug substance	Concentration [ng/mL] in 100 mg of brain tissue		
	Bulk	Extraction of nanocarriers	Direct injection of tissue with nanoparticles
Piracetam	2.8–1.0	0.25–0.01	440–275
Pentoxifylline	6000–2000	2.5–0.75	44000–20600^*^
Pyridoxine	n.d	n.d	—

^*^Predicted based on the ratio bulk/direct injection of piracetam.
